# Human fascioliasis by *Fasciola hepatica*: the first case report in Nepal

**DOI:** 10.1186/s13104-017-2761-z

**Published:** 2017-09-05

**Authors:** Ranjit Sah, Shusila Khadka, Mohan Khadka, Dipesh Gurubacharya, Jeevan Bahadur Sherchand, Keshab Parajuli, Niranjan Prasad Shah, Hari Prasad Kattel, Bharat Mani Pokharel, Basista Rijal

**Affiliations:** 1 Department of Microbiology, Institute of Medicine, Tribhuvan University and Teaching Hospital (TUTH), Kathmandu, Nepal; 2 Department of Medicine (Gastroenterology), Institute of Medicine, Tribhuvan University and Teaching Hospital (TUTH), Kathmandu, Nepal

**Keywords:** *Fasciola hepatica*, Fascioliasis, Right hypochondriac pain, TUTH (Tribhuvan University Teaching Hospital), Nepal

## Abstract

**Background:**

Fascioliasis is a zoonotic disease caused by *Fasciola* species. Patient may be asymptomatic or presents with jaundice and biliary colic or right hypochondriac pain due to bile duct obstruction with gastrointestinal symptoms.

**Case presentation:**

We report a case of human fascioliasis in a 45 years old female presented to Tribhuvan University Teaching Hospital (TUTH), Kathmandu, Nepal on August, 2015 with fever, right hypochondriac pain, jaundice and occasional vomiting with anorexia for 4 months whose alkaline phosphatase was elevated and peripheral blood smear revealed eosinophilia. The patient also gives the history of consumption of water-cress. Endoscopic Retrograde Cholagiopancretography (ERCP) showed the presence of a flat worm resembling *Fasciola hepatica* and stool routine examination revealed ova of *F. hepatica*. The patient was treated with nitazoxanide by which she got improved. Repeat stool examination 2 weeks after treatment revealed no ova of *F. hepatica*.

**Conclusions:**

Patient with fascioliasis can be simply diagnosed with stool routine microscopy and treated with nitazoxanide. So patient with right hypochondriac pain, sign and symptoms of obstructive jaundice, eosinophilia and history of water-cress consumption should be suspected for fascioliasis and investigated and treated accordingly.

## Background

Fascioliasis is caused by infection of trematodes belonging to the genus *Fasciola* (*F. hepatica* and *F. gigantica*). Its infection is known to cause bile duct inflammation and biliary obstruction [1, 2]. Fascioliasis can be presented as various clinical manifestation from mild to severe in nature. Patient may be asymptomatic or presents as gastrointestinal symptoms, chronic cholecystitis, cholangitis and liver abscesses which may be accompanied by biliary colic, epigastric pain, jaundice, pruritus and upper right quadrant pain [3]. Patient of fascioliasis often give the history of consumption of water-cress, a water plant which *F. hepatica* requires for completion of its life cycle. Many other aquatic plants including like water caltrops, water lettuce, mint and parsley have also been associated with completion of the life cycle. Individuals can also be infected by drinking water containing viable metacercariae [2].

## Case presentation

**Fig. 1:**
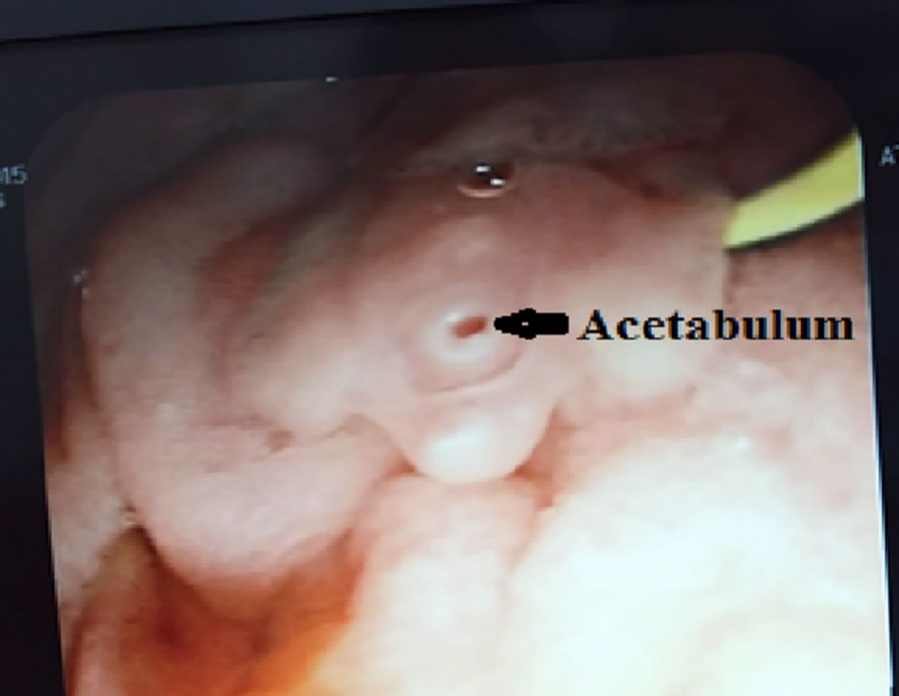
Adult worm of *Fasciola hepatica* during ERCP

**Fig. 2:**
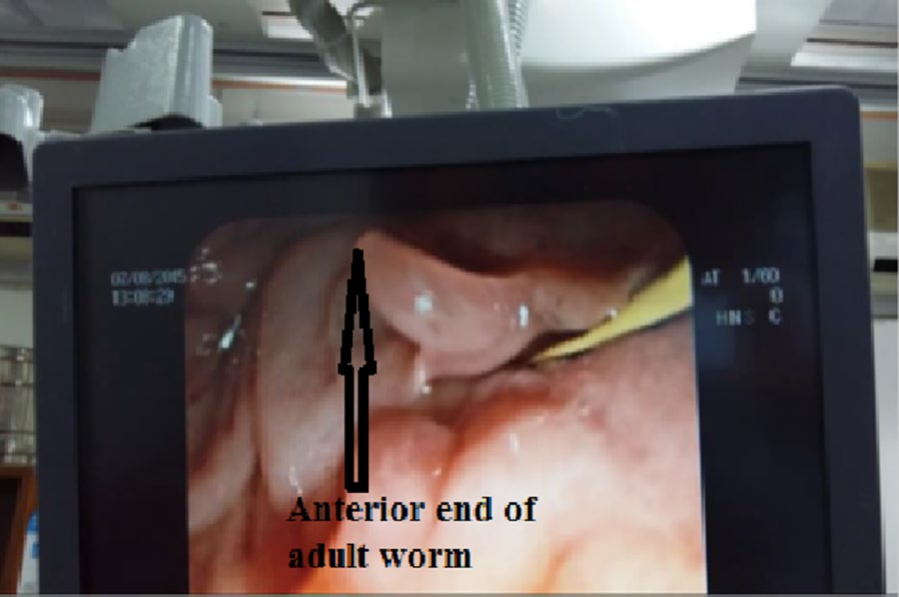
Adult worm of *Fasciola hepatica* during ERCP

We are reporting a case of 45 years old female from Surkhet presented to the medicine department of TUTH on August, 2015 with the complain of fever for 4 months which was irregular in nature, pain in the right hypochondriac region with jaundice and occasional vomiting with anorexia for same duration. She gave the history of drinking local river water with the habit of eating aquatic plant (water-cress), there was no history of consumption of raw fish. She had visited to different health institute of Nepal as well as India for seeking proper treatment, however no proper diagnosis was done and finally she was admitted in department of medicine (gastroenterology) of TUTH, Kathmandu, Nepal. A complete blood count was performed that revealed eosinophilia (Eosinophil’s: 25%, Neutrophils: 53%, Lymphocytes: 18%, Monocytes: 4% and Basophils: 0%). Total leucocytes count was 13,120/μL of blood and platelet count 325,000/μL of blood. Her hemoglobin was reduced to 7% and packed cell volume (PCV) was 22.3%. Her liver function test revealed an elevated alkaline phosphatase level (679 U/L) and gamma-glutamyl transferase (121 U/L) but normal ALT (17 U/L) and AST (10 U/L) with reduced albumin level (22 g/L). So, ERCP was done to evaluate the cause of her right hypochondriac pain, eosinophilia, an elevated alkaline phosphatase level by which an uncommon morphology of an adult worm was seen in common bile duct (Figs. 1, 2).

**Fig. 3:**
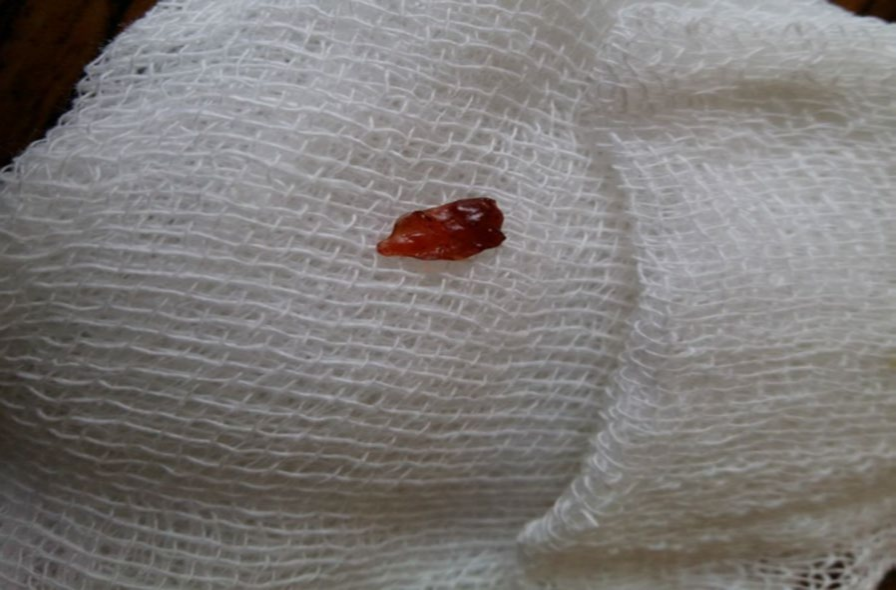
Dead adult worm of *Fasciola hepatica*

**Fig. 4:**
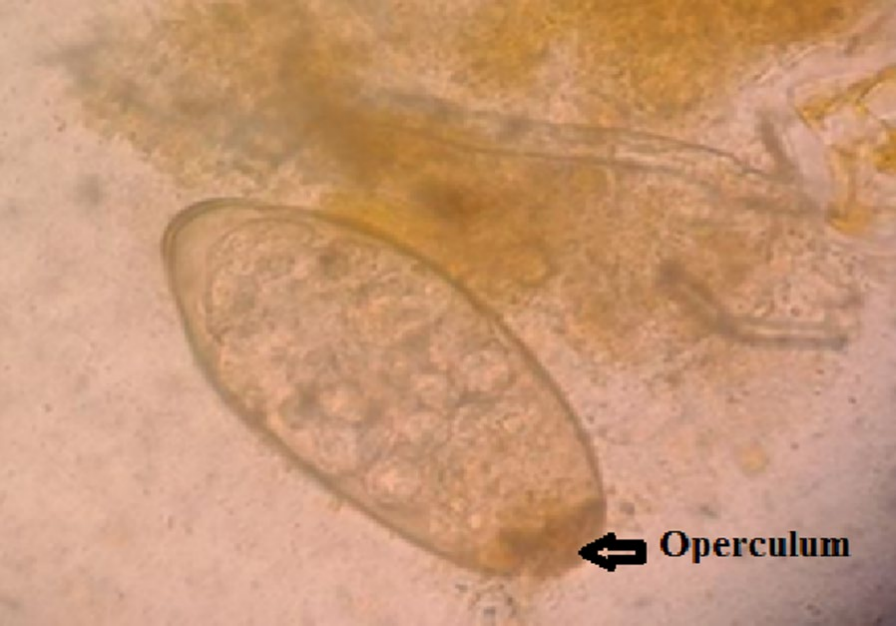
Ova of *Fasciola hepatica*

**Fig. 5:**
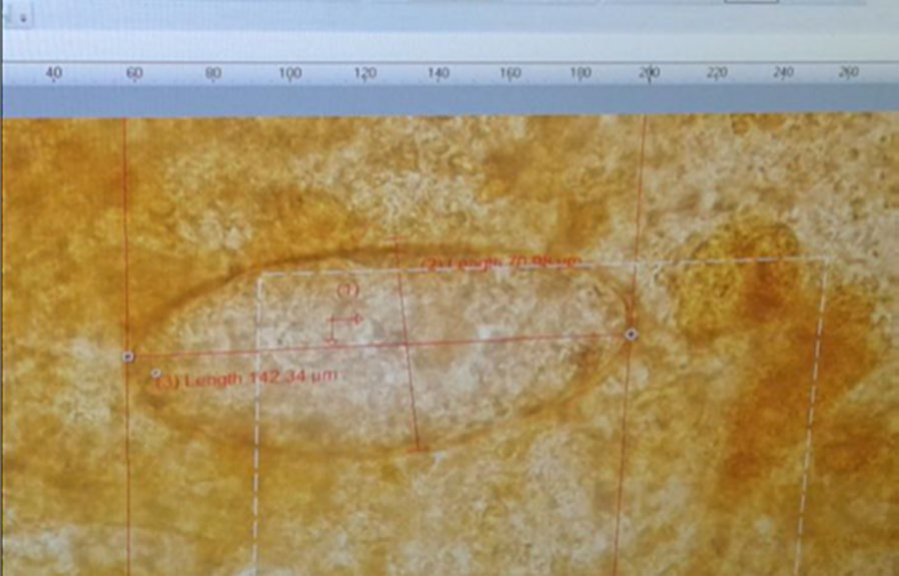
Measurement of the ova (142 μm by 70 μm) using cell sensation software version 1.12 for DP73 camera installed to the Olympus BX53 microscope used for the microscopy

The morphology of the adult worm revealed flat, leaf like measuring approximately 2–2.5 cm in length by 1 cm in width and brown to pale grey in color (Fig. 3). It had a distinct conical projection at the anterior end and broadly pointed posterior end. Patient’s stool sample was collected and processed for routine macroscopic and microscopy examination. For increased yield of ova, stool was concentrated by modified zinc sulphate concentration technique [4] and wet mount was prepared for microscopy. On macroscopic examination of stool, it was yellowish–brown with soft consistency. Microscopic examination of the wet mount of the stool sample showed large, elliptical to oval, operculated, light yellowish brown ova (Fig. 4) measuring 140–142 μm by 70–75 μm (Fig. 5). The size of the detected ova was measured using cell sensation software version 1.12 for DP73 camera installed to the Olympus BX53 microscope used for the microscopy. On the basis of morphological appearance of adult worm and characteristic feature of the detected ova and its measurement, *F. hepatica* was identified. The photographic evidence of the ova was sent to Centre for Disease Control and prevention (CDC), Atlanta, USA for confirmation and it was confirmed as ova of *F. hepatica* by CDC, Atlanta. Patient was treated with nitazoxanide 500 mg twice daily for 7 days. On third of treatment she developed high fever due to immunological reaction against the toxic product liberated by the dead worms and was managed with steroids. Follow up stool examination 2 weeks after treatment revealed no ova of *F. hepatica.*

## Discussion and conclusions

To our knowledge, this is the first report of a *F. hepatica* infection in a human in Nepal, although fascioliasis is regarded as one of the most important platyhelminthic infection of Asia and Africa [5]. Its infection is known to cause biliary tract inflammation and obstruction. Symptoms may include fever, malaise, fatigue, anorexia, weight loss and peripheral eosinophilia. Symptoms may be absent in case of light infection. Infection is more common in indigenous people and farmers who share same water sources with their animals such as sheep, goat and cattle which are the definitive host including humans and also commonly consume fresh-water aquatic plants such as water cresses [5] locally called seem-saag in Nepal that may harbor the encysted cercariae released from the snails [2].

*Fasciola hepatica* passes its life cycle in two different hosts. Sheep, goat, cattle or man are the definitive host. The adult worms reside in the biliary passage of the definitive host. The eggs are passed out in the faeces of the definitive host which in water develop into a ciliated miracidium that finds its way into the intermediate host, the *Lymnaea* snails. The miracidium passes through the stages of sporocyst. Two generations of rediae and finally to the stage of cercariae; the whole cycle taking a period of 30–60 days. The mature cercariae escape from the snail into the water and encyst in the blades of grasses or water cress. The encysted cerceriae are swallowed along with the grass and water cress by definitive host including humans. On reaching the duodenum, excystation occurs, migrate through the intestinal wall into the peritoneal cavity, penetrate the capsule of the liver, traverse its parenchyma and settle in the biliary passage. The eggs are passed in the faeces through the bile in 3–4 months of infection.

In fascioliasis, the causative agent could be *F. hepatica* or *F. gigantica.* In countries where both species co-exist, size and shape of the eggs passed in the feces are crucial diagnostic feature [5]. Eggs are identified more easily if the specimens are concentrated. Multiple specimens may need to be examined because egg production is relatively low and egg excretion may be intermittent. Negative fecal specimens do not exclude the diagnosis [6]. Serology usually becomes positive during the early phase of migration through the liver, and thus is useful in diagnosing early symptoms prior to the appearance of eggs in the feces. However, although successful treatment often correlates with a decline in antibody titers, antibodies may be detectable for years after infection [7]. So, serodiagnosis may be inconclusive. The differentiation between *F. hepatica* and *F. gigantica* infection in humans is very important because of their different transmission and epidemiological characteristics [8]. Species of the freshwater snails from the family Lymnaeidae are well known for their role as intermediate hosts in the life cycle of *Fasciola* species. The most important intermediate host for *F*. *gigantica* is *Radix auricularia*. However, other species are also known to harbour the fluke including *Lymnaea rufescens* and *L. acuminata* in the Indian Subcontinent; *Radix rubiginosa* and *R. natalensis* in Malaysia and in Africa respectively. The most important and widespread (Europe, Asia, Africa and North America) intermediate host of *F. hepatica* is *L. truncatula* [9]. Fascioliasis has a patchy distribution, with foci being related to the local distribution of intermediate snail host populations in freshwater bodies [10]. The recommended anti-parasitic agent for *F. hepatica* is triclabendazole 10 mg/kg body weight as a single dose [1]. However, we have treated our patient with nitazoxanide 500 mg twice daily for 7 days as triclabendazole is not available in Nepal and nitazoxanide is an alternative choice [1]. The patient was improved with the treatment and follow up stool routine examination 2 weeks after treatment revealed no ova of *F. hepatica*. According to Favennec et al., nitazoxanide is well-tolerated and has cure rate of 40% in children and 60 percent in adults [11]. So, treatment should be repeated if radiologic findings or eosinophilia fail to resolve or titers of serologic tests do not decrease.

Parasitic infestations are common in developing countries. However, they are wrongly diagnosed as other medical or surgical conditions and remain untreated for long. Infections like fascioliasis can be diagnosed by simple stool routine microscopy examination and their treatment is simply a short course of anti-helminthic therapy. So, patient with supporting clinical history and clinical findings should be suspected and searched for the evidence of such parasitic infections. Family members of the patient should also be screened for the infection as they may harbor the parasite with or without symptoms since they share the common food and water.

## Abbreviations

*F. hepatica*: *Fasciola hepatica*
*F. gigantica*: *Fasciola gigantica*
TUTH: Tribhuvan University Teaching Hospital
ERCP: Endoscopic Retrograde Cholangiopancreatography
CDC: Centre of Disease Control and Prevention
USA: United States of America
µL: microlitre
mg: milligram
g/L: gram per litre
U/L: unit per litre
PCV: packed cell volume
ALT: alanine aminotransferase
AST: aspartate aminotransferase
